# Impact of diagnostic labels and management benefit to harms information for low-risk prostate pathology: Two online factorial, randomized, hypothetical scenario experiments in Australian males and female partners

**DOI:** 10.1093/jnci/djag248

**Published:** 2026-07-24

**Authors:** Katy J L Bell, Farzaneh Boroumand, Zhuohan Wu, Timothy J Wilt, Lisa Parker, Jeremy Millar, Jenna Smith, Kirsten McCaffery, John Brandt Brodersen, Philipp Dahm, Brett Delahunt, Paul Glasziou, Andrew Warden, Lawrence Diller, Christo van Rensburg, Brooke Nickel

**Affiliations:** Sydney School of Public Health, Faculty of Medicine and Health, The University of Sydney, Sydney, NSW, Australia; Wiser Healthcare Research Collaboration, University of Sydney, Sydney, NSW, Australia; Sydney School of Public Health, Faculty of Medicine and Health, The University of Sydney, Sydney, NSW, Australia; Sydney School of Public Health, Faculty of Medicine and Health, The University of Sydney, Sydney, NSW, Australia; Wiser Healthcare Research Collaboration, University of Sydney, Sydney, NSW, Australia; Minneapolis VA Center for Care Delivery and Outcomes Research, Minneapolis, MN, United States; School of Medicine and School of Public Health, University of Minnesota, Minneapolis, MN, United States; Northern Clinical School, Faculty of Medicine & Health, Charles Perkins Centre, University of Sydney, Sydney, NSW, Australia; Department of Radiation Oncology, Royal North Shore Hospital, Sydney, NSW, Australia; School of Public Health and Preventive Medicine, Monash University, Melbourne, VIC, Australia; Radiation Oncology, Alfred Health, Melbourne, VIC, Australia; Sydney School of Public Health, Faculty of Medicine and Health, The University of Sydney, Sydney, NSW, Australia; Wiser Healthcare Research Collaboration, University of Sydney, Sydney, NSW, Australia; Sydney School of Public Health, Faculty of Medicine and Health, The University of Sydney, Sydney, NSW, Australia; Wiser Healthcare Research Collaboration, University of Sydney, Sydney, NSW, Australia; Zealand University Hospital, Copenhagen, Denmark; Department of People and Technology, Roskilde University, Roskilde, Denmark; Research Unit for General Practice, UiT The Arctic University of Norway, Tromsø, Norway; Urology Section, Minneapolis VA Health Care System, Minneapolis, MN, United States; Department of Urology, University of Minnesota, Minneapolis, MN, United States; Malaghan Institute of Medical Research, Wellington, New Zealand; Department of Oncology-Pathology, Karolinska Institute, Stockholm, Sweden; Wiser Healthcare Research Collaboration, University of Sydney, Sydney, NSW, Australia; Institute for Evidence-Based Healthcare, Faculty of Health Sciences and Medicine, Bond University, Gold Coast, QLD, Australia; Wiser Healthcare Research Collaboration, University of Sydney, Sydney, NSW, Australia; Wiser Healthcare Research Collaboration, University of Sydney, Sydney, NSW, Australia; Health Consumers New South Wales, Sydney, NSW, Australia; Sydney School of Public Health, Faculty of Medicine and Health, The University of Sydney, Sydney, NSW, Australia; Wiser Healthcare Research Collaboration, University of Sydney, Sydney, NSW, Australia

## Abstract

**Background:**

We sought to evaluate the effects of diagnostic labels and management benefit to harms information for low-risk prostate pathology.

**Methods:**

Two factorial (3 × 2) online hypothetical experiments randomly assigned participants 1:1:1 to 3 labels: *low-risk prostate cancer, Gleason group 1*; *low-risk prostate neoplasm*; or *low-risk prostate lesion*, with second random assignment 1:1 to high or low information about management options. Participants were Australians at least 50 years of age with a prostate (male individuals) or with a male partner aged at least 50 years (female individuals). The primary outcome was preference for management with curative intent (prostatectomy, radiation therapy) or conservative management (active surveillance, prostate-specific antigen monitoring).

**Results:**

For 1340 males and 1435 females randomly assigned April-June 2024, 50% vs 34% preferred prostate-specific antigen monitoring, 37% vs 46% preferred active surveillance, 7% vs 11% preferred prostatectomy, and 7% vs 8% preferred radiation therapy. The *neoplasm* label reduced the preference for curative-intent treatment in males (low information risk difference  = ‒3.9%, 95% CI = ‒10.5% to 2.7%; high information risk difference = ‒5.2%, 95% CI = ‒10.6% to 0.2%) and females (low information risk difference = ‒6.6%, 95% CI = ‒14.3% to 1.1%; high information risk difference = ‒2.3%, 95% CI = ‒8.9% to 4.3%). The *lesion* label reduced the preference for curative-intent treatment among females in low information groups (risk difference = ‒11.6%, 95% CI = ‒18.9% to ‒4.3%) but had minimal effect in males and in females in high information groups. High information reduced the preference for curative-intent treatment within the *cancer* label groups in both studies (risk difference among males = ‒4.0%, 95% CI = ‒10.5% to 2.5%; risk difference among females = ‒10.3%, 95% CI = ‒17.7% to ‒2.9%). The combination of *neoplasm* and high information resulted in the largest effects (risk difference among males = ‒9.2%, 95% CI = ‒15.1% to ‒3.3%; risk difference among females = ‒12.6%, 95% CI = ‒19.9% to ‒5.2%).

**Conclusions:**

Clear communication about management benefit to harm information and using *neoplasm* to describe low-risk prostate pathology may support conservative management choices and mitigate overtreatment.

**Trial registration:**

ANZCTR 386701; 386889.

## Introduction

To mitigate harms from overdiagnosis and overtreatment of low-risk prostate cancer,^1^^,^^2^ conservative management is recommended through active surveillance, including prostate-specific antigen (PSA) tests, magnetic resonance imaging scans, and routine biopsies, or PSA monitoring (PSA tests alone), with delayed treatment with curative intent if there is evidence of disease progression.^3^ A barrier to uptake is the perception that all prostate cancers cause morbidity and mortality if not immediately treated.^4^ Potential solutions to this barrier include (1) use of an alternative diagnostic label that avoids the word *cancer*^5-7^ to help reduce anxiety and (2) better communication about absolute benefits and harms of curative-intent treatment and conservative management strategies.^8^

We tested both of these potential solutions through online factorial randomized studies in Australians with a prostate but no history of prostate cancer (males) and Australians without a prostate but with a partner who has a prostate (females). We included female partners because they are often involved in prostate cancer treatment decisions, and the diagnosis can have negative impacts on their mental and physical well-being.^9^ Patients and their partners may perceive all prostate cancers as being fatal or causing serious morbidity if not immediately treated.^4^ We evaluated the effects of 2 alternative diagnostic labels for a hypothetical low-risk prostate diagnosis (*neoplasm* and *lesion*) compared with a control label (*cancer*) and the provision of absolute risk information about benefits and harms of management options (high information) compared with only describing the management options (low information).

## Methods

### Reporting

We followed the CONSORT extension for factorial randomized trials guideline^10^^,^^11^ and the Guideline for RepOrting Vignette Experiments.^12^

### Protocol

The protocol was approved by Human Research Ethics Committee at the University of Sydney (2023/572) and published.^13^ The study was registered in the Australian New Zealand Clinical Trials Registry (identifiers 386701 and 386889).

### Trial design

The 3 × 2 factorial, randomized, hypothetical scenario–based online experiments evaluated diagnostic labels’ effects (*neoplasm* and *lesion* vs *cancer*) and information quality about treatments (high information vs low information) on preference for curative-intent treatment (prostatectomy or radiation therapy [RT]) compared with conservative management (PSA-based monitoring or active surveillance). After providing written consent, participants responded to questions on sociodemographic and clinical characteristics, were randomly assigned to 1 of 6 hypothetical scenarios simulating a clinic appointment with their general practitioner after a prostate biopsy to investigate an elevated PSA result for themselves or their partner, and responded to questions measuring outcomes.

### Participants

Participants recruited by a market research provider Qualtrics from among a prearranged pool of respondents who agreed to be contacted for survey responses.^14^ These participants were Australian residents, were males aged at least 50 years or females aged at least 30 years with a male partner aged at least 50 years, had no history (males) or partner history (females) of prostate cancer, and were able to understand written English.

### Setting and location

The online study collected data using Qualtrics survey software (Qualtrics, Provo, UT, 2020).

### Interventions

Participants were told that they (males) or their partner (females) had received a particular diagnosis (Box S1) and were given information about management options (Box S2, Figure S1). In the low information (control) condition, participants were provided with a description of the 4 management options and told that different treatments have different advantages and disadvantages (Box S2). In the high information condition, they were additionally presented with icon array graphical display of the probability of problems with erectile dysfunction, bladder control, and bowel control as well as the risk of distant metastasis after 15 years (Figure S1). The 2 alternative diagnostic labels were chosen by the clinician, patient, and public co-investigators through an iterative co-design process using online surveys, described in detail in the protocol.^13^ The 3 levels for the diagnostic label factor were (1) low-risk prostate cancer, Gleason Group 1 (control); (2) low-risk prostate neoplasm (alternative label 1); and (3) low-risk prostate lesion (alternative label 2). The 2 information levels were (1) short description of diagnosis and 4 management options (control) or (2) information about the absolute probability of erectile, urinary, and bowel function and metastatic spread for each management option (as reported in the Prostate Cancer Intervention Versus Observation Trial [PIVOT]^15-17^ and Prostate Testing for Cancer and Treatment (ProtecT) randomized clinical trials [RCTs]^18-20^) presented as icon arrays (Figure S1).

### Outcomes

Box S3 provides an overview of the prespecified primary and secondary outcomes and covariates.^13^ The primary outcome was preferred management option: curative-intent treatment (prostatectomy or RT) vs conservative management (active surveillance or PSA monitoring). Secondary outcomes were preference for each individual management option, diagnosis anxiety,^21^ management choice anxiety,^21^ open-text explanation of management preference (free-text input; findings presented in a separate report), and whether the participant would prefer curative-intent treatment after 5 years of conservative management (for participants who preferred these options initially).

### Sample size

We estimated that 1290 males (430 participants per diagnostic label group) and 1410 females (470 participants per diagnostic label group) would provide 80% power to detect a pairwise difference in the absolute proportion preferring curative-intent treatment, between the *cancer* label group and each alternative label group, as small as 10% within each study. This calculation assumed no interaction between the diagnostic label and information factors; that 30% of males and 35% of female partners^22^^,^^23^ would prefer curative-intent treatment in the control label; a 5% dropout rate; α = .05; the normal approximation to the binomial distribution; and the standard formula for comparing proportions in independent, equal-sized groups.^24^

### Random assignment, allocation concealment, implementation, and blinding

Qualtrics uses the Mersenne Twister pseudorandom number generator to randomly assign participants. We used recruitment quotas for education, states and territories, and age. The online survey design ensured that allocation sequence was concealed from participants and researchers. Participants were unblinded after intervention assignment. Researchers remained blinded through data analysis.

### Statistical methods

We defined 4 estimands of interest (Box S4) and used multiarm analysis as our primary analytic approach.^11^ Relevant contrasts were defined to compare label groups (label 1, label 2, control) and information groups (high, low), according to the 4 estimands of interest. We calculated absolute risks and corresponding risk differences with 95% CIs as well as mean levels and difference in means with 95% CIs directly from the observed data. Differences in proportions between males and female partners were assessed using 2-sample tests for equality of proportions (ꭓ^2^ test), and 95% CIs for the absolute difference in proportions were calculated. For the primary outcome, we also built multivariable logistic regression models to calculate *P* values for intervention effects, adjusting for potential prognostic factors measured at baseline to improve precision.^25^^,^^26^ Models were adjusted for covariates prespecified a priori (Box S3). All analyses were conducted in R, version 4.4.2, statistical software (R Foundation for Statistical Computing), using the *nnet, emmeans, broom*, and *gtsummary* packages.

## Results

Between April 11 and June 5, 2024, 9364 people were assessed for eligibility; 1402 people were randomly assigned in the male study and 1493 people in the female study (Figure 1). Of those individuals randomly assigned, 62 males and 58 females did not finish the survey and were excluded from analysis. Tables S1 and S2 show baseline demographic and clinical characteristics of included males (*n* = 1340) and females (*n* = 1435). For males vs females, the proportion that preferred conservative management was 86.9% (1165/1340) vs 81.0% (1162/1435) (risk difference = 6.0%, 95% CI = 3.2% to 8.7%; *P* < .001). The male vs female proportions that preferred specific options were PSA monitoring, 49.7% (666/1340) vs 34.5% (495/1435) (risk difference = 15.2%, 95% CI = 11.6% to 18.8%; *P* < .001); active surveillance, 37.2% (499/1340) vs 46.5% (667/1435) (risk difference = ‒9.2%, 95% CI = ‒12.9% to ‒5.6%; *P* < .001); prostatectomy, 6.6% (88/1340) vs 10.8% (155/1435) (risk difference = ‒4.2%, 95% CI = ‒6.3% to ‒2.2%; *P* < .001); and RT, 6.5% (87/1340) vs 8.2% (118/1435) (risk difference = ‒1.7%, 95% CI = ‒3.6% to 0.3%; *P* = .10).

**Figure 1.**
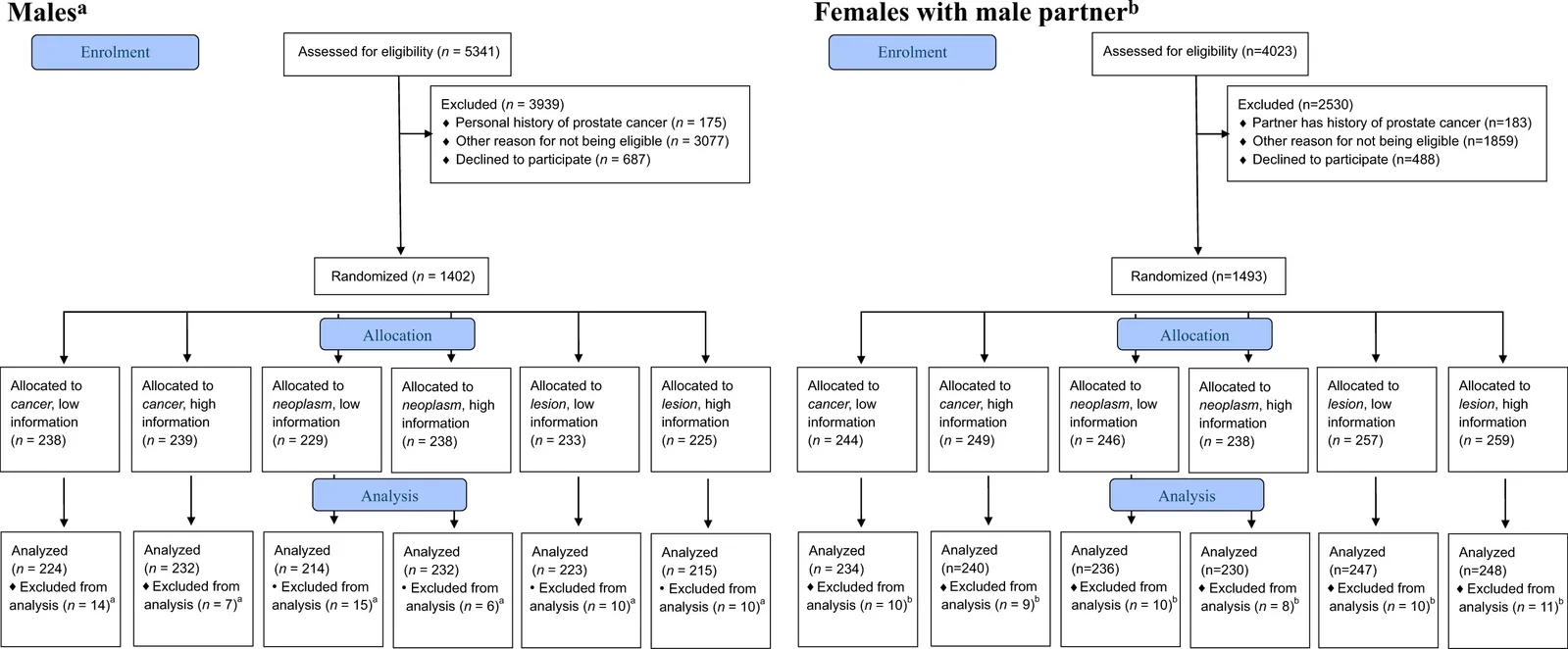
Recruitment and analysis of Australian participants in 2 randomized online studies of labels for low-risk prostate pathology. ^a^We excluded 62 participants from our analysis of the Australian male study because they did not finish the survey. ^b^We excluded 58 participants from our analysis of the Australian female partner study because they did not finish the survey.

### Primary outcome: preference for curative intent vs conservative management

Label effects for low and high information settings are summarized in Figure 2. There was evidence of interactions between label and information factors in males (*P* = .02) and female partners (*P* = .009). Both alternative labels had small and uncertain effects in the male low information groups (Table 1). Compared with 16.5% (37/224) of those assigned *cancer*, 12.6% (27/214) assigned *neoplasm* (risk difference = ‒3.9%, 95% CI = ‒10.5% to 2.7%) and 14.3% (32/223) assigned *lesion* (risk difference = ‒2.2%, 95% CI = ‒8.9% to 4.5%) preferred curative-intent treatment. These effects were larger in the female low information groups (Table 2). Compared with 27.4% (64/234) of those assigned *cancer*, 20.8% (49/236) assigned *neoplasm* (risk difference = ‒6.6%, 95% CI = ‒14.3% to 1.1%) and 15.8% (39/247) assigned lesion (risk difference = ‒11.6%, 95% CI = ‒18.9% ‒4.3%) preferred curative-intent treatment.

**Figure 2.**
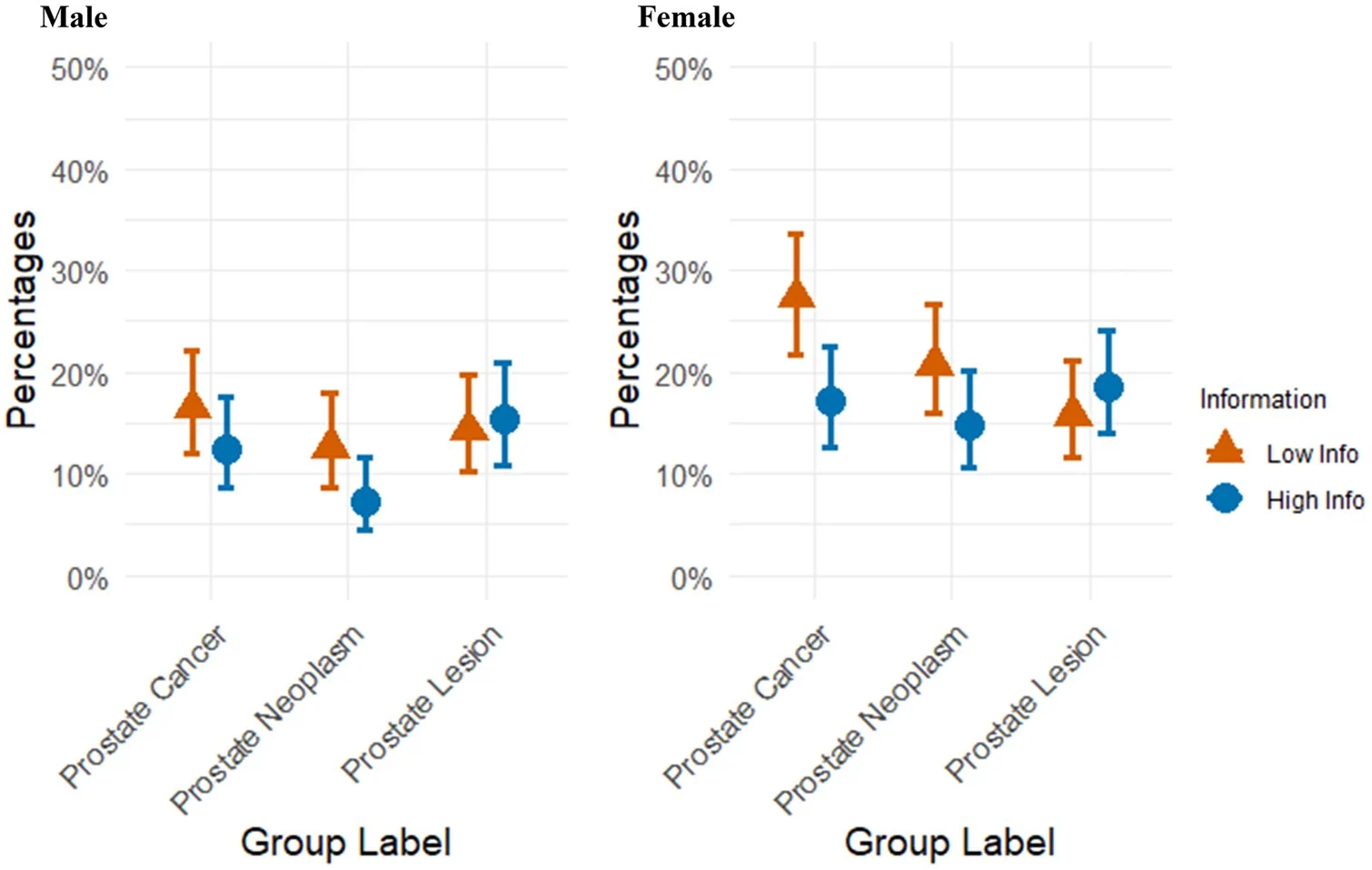
Proportions preferring curative-intent treatment (prostatectomy or radiation therapy) over conservative management (prostate-specific antigen monitoring or active surveillance) for management of low-risk prostate pathology. Likelihood ratio tests in the multivariable models indicated statistically significant interactions between label and information factors in men (*P* = .02) and women (*P* = .009).

**Table 1.** Diagnostic label effects among males across low information groups (estimand 1: usual care comparator).

Outcome	Prostate *cancer n* = 224	Prostate *neoplasm n* = 214	Absolute difference (95% CI)^1^	Prostate *lesion n* = 223	Absolute difference, % (95% CI)
Prefer curative-intent treatment, No. (%)	37 (16.5)	27 (12.6)	Risk difference = ‒3.9% (‒10.5 to 2.7)^a^	32 (14.3)	Risk difference = ‒2.2 (‒8.9 to 4.5)^b^
Prefer prostatectomy, No. (%)	18 (8.0)	14 (6.5)	Risk difference = ‒1.5 (‒6.4 to 3.4)	16 (7.2)	Risk difference = ‒0.9 (‒5.8 to 4.0)
Prefer RT, No. (%)	19 (8.5)	13 (6.1)	Risk difference = ‒2.4 (‒7.3 to 2.4)	16 (7.2)	Risk difference = ‒1.3 (‒6.3 to 3.7)
Prefer active surveillance, No. (%)	83 (37.1)	83 (38.8)	1.7 (‒7.4 to 10.8)	90 (40.4)	Risk difference = 3.3 (‒5.7 to 12.3)
Prefer PSA monitoring, No. (%)	104 (46.4)	104 (48.6)	Risk difference = 2.2 (‒7.2 to 11.5)	101 (45.3)	Risk difference = ‒1.1 (‒10.4 to 8.1)
Diagnosis anxiety (visual analog scale, 1-10), mean (SD)	4.97 (2.41)	5.00 (2.62)	Mean difference = 0.03 (‒0.45 to 0.50)	4.66 (2.59)	Mean difference = ‒0.31 (‒0.78 to 0.15)
Management anxiety (visual analog scale, 1-10), mean (SD)	4.45 (2.32)	4.38 (2.50)	Mean difference = ‒0.07 (‒0.52 to 0.39)	4.08 (2.51)	Mean difference = ‒0.38 (‒0.82 to 0.07)
After 5 y of surveillance or monitoring, chose curative surgery without medical indication^c^, No. (%)	21 (11.2)	24 (12.8)	Risk difference = 1.6 (‒5.0 to 8.2)	27 (14.1)	Risk difference = 2.9 (‒3.8 to 9.6)

Abbreviations: PSA = prostate-specific antigen; RT = radiation therapy.

a *P* = .13 for *prostate neoplasm* label effect on preference for curative-intent treatment in multivariable regression model.

b *P* = .56 for *prostate lesion* label effect on preference for curative-intent treatment in multivariable regression model.

c Percentages are for those participants who did not choose initial curative treatment (ie, denominators are those who chose active surveillance or PSA monitoring).

**Table 2.** Diagnostic label effects in female partners across low information groups (estimand 1: label effects with usual care comparator).

Outcome	Prostate *cancer n* = 234	Prostate *neoplasm n* = 236	Absolute difference (95% CI)	Prostate *lesion n* = 247	Absolute difference (95% CI)
Prefer curative-intent treatment, No. (%)	64 (27.4)	49 (20.8)	Risk difference = ‒6.6 (‒14.3 to 1.1)^a^	39 (15.8)	Risk difference = ‒11.6 (‒18.9 to ‒4.3)^b^
Prefer prostatectomy, No. (%)	41 (17.5)	30 (12.7)	Risk difference = ‒4.8 (‒11.3 to 1.6)	24 (9.7)	Risk difference = ‒7.8 (‒13.9 to ‒1.7)
Prefer RT, No. (%)	23 (9.8)	19 (8.1)	Risk difference = ‒1.8 (‒6.9 to 3.4)	15 (6.1)	Risk difference = ‒3.8 (‒8.6 to 1.1)
Prefer active surveillance, No. (%)	100 (42.7)	112 (47.5)	Risk difference = 4.7 (‒4.3 to 13.7)	122 (49.4)	Risk difference = 6.7 (‒2.2 to 15.6)
Prefer PSA monitoring, No. (%)	70 (29.9)	75 (31.8)	Risk difference = 1.9 (‒6.5 to 10.2)	86 (34.8)	Risk difference = 4.9 (‒3.4 to 13.2)
Diagnosis anxiety (visual analog scale, 1-10), mean (SD)^c^	6.30 (2.27)	6.13 (2.32)	Mean difference = ‒0.17 (‒0.59 to 0.24)	6.00 (2.25)	Mean difference = ‒0.30 (‒0.70 to 0.11)
Management anxiety (visual analog scale, 1-10), mean (SD)	5.37 (2.31)	5.20 (2.31)	Mean difference = ‒0.16 (‒0.58 to 0.25)	5.09 (2.16)	Mean difference = ‒0.27 (‒0.68 to 0.13)
After 5 y, chose curative surgery without medical indication^d^, No. (%)	21 (12.4)	22 (11.8)	Risk difference = ‒0.6 (‒7.4 to 6.2)	28 (13.5)	Risk difference = 1.1 (‒5.7 to 7.9)

Abbreviations: PSA = prostate-specific antigen; RT = radiation therapy.

a *P* = .38 for prostate *neoplasm* label effect on preference for curative-intent treatment in multivariable regression model.

b *P* = .008 for prostate *lesion* label effect on preference for curative-intent treatment in multivariable regression model.

c Missing outcome for 1 person in the prostate *cancer* group, 2 people in the prostate *neoplasm* group, and 1 person in the prostate *lesion* group.

d Percentages are for those participants who did not choose initial curative treatment (ie, denominators are those who chose active surveillance or PSA monitoring).

The alternative labels had uncertain effects that were in opposing directions in the male high information groups (Table S3). Compared with 12.5% (29/232) of the *cancer* group, 7.3% (17/232) of the *neoplasm* group (risk difference = ‒5.2%, 95% CI = ‒10.6% to 0.2%), and 15.3% (33/215) of the *lesion* group (risk difference = 2.8%, 95% CI = ‒3.6% to 9.2%) preferred curative-intent treatment (Table S3). We observed the same pattern in the female high information groups (Table S4). Compared with 17.1% (41/240) of the *cancer* group, 14.8% (34/230) of the *neoplasm* group (risk difference = ‒2.3%, 95% CI = ‒8.9% to 4.3%), and 18.5% (46/248) of the *lesion* group (risk difference = 1.4%, 95% CI = ‒5.4% to 8.2%) preferred curative-intent treatment (Table S4).

The benefit-harm information effect was small and uncertain in male *cancer* label groups (Table 3). Compared with 16.5% (37/224) of those assigned to low information, 12.5% (29/232) assigned high information (risk difference = ‒4.0%, 95% CI = ‒10.5% to 2.5%) preferred curative-intent treatment. This effect was larger in female *cancer* label groups (Table 4). Compared with 27.4% (64/234) of those assigned to low information, 17.1% (41/240) assigned to high information (risk difference = ‒10.3%, 95% CI = ‒17.7% to ‒2.9%) preferred curative-intent treatment.

**Table 3.** Information effects in males across *cancer* diagnostic label groups (estimand 3: label effects with usual care comparator).

Outcome	Low information *n* = 224	High information *n* = 232	Absolute difference (95% CI)
Prefer curative-intent treatment, No. (%)	37 (16.5)	29 (12.5)	Risk difference = ‒4.0 (‒10.5 to 2.5)^a^
Prefer prostatectomy, No. (%)	18 (8.0)	12 (5.2)	Risk difference = ‒3.0 (‒7.0 to 2.0)
Prefer RT, No. (%)	19 (8.5)	17 (7.3)	Risk difference = ‒1.0 (‒6.0 to 4.0)
Prefer active surveillance, No. (%)	83 (37.1)	79 (34.1)	Risk difference = ‒3.0 (‒12.0 to 6.0)
Prefer PSA monitoring, No. (%)	104 (46.4)	124 (53.4)	Risk difference = 7.0 (‒2.0 to 16.0)
Diagnosis anxiety (visual analog scale, 1-10), mean (SD)	4.97 (2.41)	4.59 (2.65)	Mean difference = ‒0.38 (‒0.85 to 0.08)
Management anxiety (visual analog scale, 1-10), mean (SD)	4.45 (2.32)	4.16 (2.60)	Mean difference = ‒0.29 (‒0.74 to 0.16)
After 5 y of surveillance or monitoring, chose curative surgery without medical indication^c^, No. (%)	21 (11.2)	24 (11.8)	Risk difference = 0.6 (‒5.7 to 6.9)

Abbreviations: PSA = prostate-specific antigen; RT = radiation therapy.

a *P* = .26 for high information effect on preference for curative-intent treatment in multivariable regression model.

b Percentages are for those participants who did not choose initial curative treatment (ie, denominators are those who chose active surveillance or PSA monitoring).

**Table 4.** Information effects in female partners across *cancer* diagnostic label groups (estimand 3: information effects with usual care comparator).

Outcome	Low information *n* = 234	High information *n* = 240	Absolute difference (95% CI)
Prefer curative-intent treatment, No. (%)	64 (27.4)	41 (17.1)	Risk difference = ‒10.3 (‒17.7 to ‒2.9)^a^
Prefer prostatectomy, No. (%)	41 (17.5)	17 (7.1)	Risk difference = ‒10.4 (‒16.3 to ‒4.6)
Prefer RT, No. (%)	23 (9.8)	24 (10.0)	Risk difference = 0 (‒5.2 to 5.6)
Prefer active surveillance, No. (%)	100 (42.7)	118 (49.2)	Risk difference = 6.5 (‒2.5 to 15.4)
Prefer PSA monitoring, No. (%)	70 (29.9)	81 (33.8)	Risk difference = 3.8 (‒4.5 to 12.2)
Diagnosis anxiety (visual analog scale, 1-10), mean (SD)^b^	6.30 (2.27)	5.84 (2.43)	Mean difference = ‒0.46 (‒0.88 to ‒0.036)
Management anxiety (visual analog scale, 1-10), mean (SD)	5.37 (2.31)	5.4 (2.49)	Mean difference = 0.03 (‒0.40 to 0.47)
After 5 y of surveillance or monitoring, chose curative surgery without medical indication^c^, No. (%)	21 (12.4)	26 (13.1)	Risk difference = 0.7 (‒6.1 to 7.5)

Abbreviations: PSA = prostate-specific antigen; RT = radiation therapy.

a *P* = .007 for high information effect on preference for curative-intent treatment in multivariable regression model.

b Missing outcome for 1 person in the low information group and 1 person in the high information group.

c Percentages are for those participants who did not choose initial curative treatment (ie, denominators are those who chose active surveillance or PSA monitoring).

The combined label plus benefit-harm information effects differed by alternative label among males. Compared with 16.5% (37/224) of those assigned *cancer* plus low information, 7.3% (17/232) of those assigned *neoplasm* plus high information (risk difference = ‒9.2%, 95% CI = ‒15.1% to ‒3.3%) and 15.3% (33/215) of those assigned *lesion* plus high information (risk difference = ‒1.2%, 95% CI = ‒8.0% to 5.7%) preferred curative-intent treatment (Table 5). The same pattern was observed in females, but the effects for both labels were larger than in males, reflecting the higher proportion preferring curative-intent treatment in the comparator (*cancer* label plus low information) group. Compared with 27.4% (64/234) of those assigned *cancer* plus low information, 14.8% (34/230) of those assigned *neoplasm* plus high information (risk difference = ‒12.6%, 95% CI = ‒19.9% to ‒5.2%), and 18.5% (46/248) of those assigned *lesion* plus high information (risk difference = ‒8.9%, 95% CI = ‒16.3% to ‒1.3%) preferred curative-intent treatment (Table 6).

**Table 5.** Combined effects of diagnostic label and high information among males (estimand 4: combined label plus information effects with usual care comparator).

Outcome	Prostate *cancer* plus low information *n* = 224	Prostate *neoplasm* plus high information *n* = 232	Absolute difference (95% CI)	Prostate *lesion* plus high information *n* = 215	Absolute difference (95% CI)
Prefer curative-intent treatment, No. (%)	37 (16.5)	17 (7.3)	Risk difference = ‒9.2 (‒15.1 to ‒3.3)^a^	33 (15.3)	Risk difference = ‒1.2 (‒8.0 to 5.7)^b^
Prefer prostatectomy, No. (%)	18 (8.0)	10 (4.3)	Risk difference = ‒3.7 (‒8.1 to 0.7)	18 (8.4)	Risk difference = 0.3 (‒4.8 to 5.5)
Prefer RT, No. (%)	19 (8.5)	7 (3.0)	Risk difference = ‒5.5 (‒9.7 to ‒1.2)	15 (7.0)	Risk difference = ‒1.5 (‒6.5 to 3.5)
Prefer active surveillance, No. (%)	83 (37.1)	83 (35.8)	Risk difference = ‒1.3 (‒10.1 to 7.6)	81 (37.7)	Risk difference = 0.6 (‒8.4 to 9.7)
Prefer PSA monitoring, No. (%)	104 (46.4)	132 (56.9)	Risk difference = 10.5 (1.3 to 19.6)	101 (47.0)	Risk difference = 0.5 (‒8.8 to 9.9)
Diagnosis anxiety (visual analog scale, 1-10), mean (SD)	4.97 (2.41)	4.78 (2.68)	Mean difference = ‒0.19 (‒0.66 to 0.28)	4.67 (2.74)	Mean difference = ‒0.30 (‒0.79 to 0.18)
Management anxiety (visual analog scale, 1-10), mean (SD)	4.45 (2.32)	4.22 (2.55)	Mean difference = ‒0.23 (‒0.68 to 0.22)	4.11 (2.73)	Mean difference = ‒0.34 (‒0.81 to 0.14)
After 5 y, chose curative surgery without medical indication^c^, No. (%)	21 (11.2)	25 (11.6)	Risk difference = 0.4 (‒5.8 to 6.6)	16 (8.8)	Risk difference = ‒2.4 (‒8.6 to 3.7)

Abbreviations: PSA = prostate-specific antigen; RT = radiation therapy.

a *P* = .002 for prostate *neoplasm* label effect on preference for curative-intent treatment in multivariable regression model.

b *P* = .99 prostate *lesion* label effect on preference for curative-intent treatment in multivariable regression model.

c Percentages are for those participants who did not choose initial curative treatment (ie, denominators are those who chose active surveillance or PSA monitoring).

**Table 6.** Combined effects of diagnostic label and high information in female partners.

Outcome	Prostate *cancer* plus low information *n* = 234	Prostate *neoplasm* plus high information *n* = 230	Absolute difference (95% CI)	Prostate *lesion* plus high information *n* = 248	Absolute difference (95% CI)
Prefer curative-intent treatment, No. (%)	64 (27.4)	34 (14.8)	Risk difference = ‒12.6 (‒19.9 to ‒5.2)^a^	46 (18.5)	Risk difference = ‒8.9 (‒16.3 to ‒1.3)^b^
Prefer prostatectomy, No. (%)	41 (17.5)	17 (7.4)	Risk difference = ‒10.1 (‒16.1 to ‒4.2)	26 (10.5)	Risk difference = ‒7.0 (‒13.2 to ‒0.9)
Prefer RT, No. (%)	23 (9.8)	17 (7.4)	Risk difference = ‒2.4 (‒7.5 to 2.7)	20 (8.1)	Risk difference = ‒1.8 (‒6.9 to 3.3)
Prefer active surveillance, No. (%)	100 (42.7)	107 (46.5)	Risk difference = 3.8 (‒5.3 to 12.8)	108 (43.5)	Risk difference = 0.8 (‒8.0 to 9.7)
Prefer PSA monitoring, No. (%)	70 (29.9)	89 (38.7)	Risk difference = 8.8 (0.2 to 17.4)	94 (37.9)	Risk difference = 8.0 (‒0.4 to 16.4)
Diagnosis anxiety (visual analog scale, 1-10), mean (SD)	6.30 (2.27)	5.47 (2.48)	Mean difference = ‒0.83 (‒1.26 to ‒0.40)	5.84 (2.22)	Mean difference = ‒0.46 (‒0.86 to ‒0.06)
Management anxiety (visual analog scale, 1-10), mean (SD)	5.37 (2.31)	4.94 (2.53)	Mean difference = ‒0.43 (‒0.87 to 0.01)	5.24 (2.29)	Mean difference = ‒0.13 (‒0.54 to 0.28)
After 5 y, chose curative surgery without medical indication^c^, No. (%)	21 (12.4)	20 (10.2)	Risk difference = 0.6 (‒7.4 to 6.2)	32 (15.8)	Risk difference = 1.1 (‒5.7 to 7.9)

Abbreviations: PSA = prostate-specific antigen; RT = radiation therapy.

a *P* = .004 for prostate *neoplasm* label effect on preference for curative-intent treatment in multivariable regression model.

b *P* = .02 for prostate *lesion* label effect on preference for curative-intent treatment in multivariable regression model.

c Percentages are for those participants who did not choose initial curative treatment (ie, denominators are those who chose active surveillance or PSA monitoring).

### Secondary outcomes

#### Preference for specific conservative management options vs prostatectomy

In males, across both the low and high information groups, the effects of the *neoplasm* and *lesion* labels on the probability of preferring active surveillance or of preferring PSA monitoring were small and uncertain, with wide CIs (Table 1; Table S3). The point estimates indicated that the *neoplasm* groups had numerically higher probabilities of preferring both conservative management options. The *lesion* groups had numerically higher probabilities of preferring active surveillance but numerically lower probabilities of preferring PSA monitoring.

Label effects were larger in female low information groups, with the largest effect observed for *lesion* effect on preference for prostatectomy (Table 2). Compared with 17.5% (41/234) of those assigned to *cancer*, 9.7% (24/247) of those assigned to *lesion* preferred prostatectomy (risk difference = ‒7.8%, 95% CI = ‒13.9% to ‒1.7%). Effects in female high information groups were smaller. The point estimates indicated that both alternative label groups had numerically higher probabilities of preferring PSA monitoring and numerically lower probabilities of preferring active surveillance (Table S4).

The benefit-harm information effect was small and uncertain in males. Participants receiving high information had a numerically higher probability of preferring PSA monitoring and a numerically lower probability of preferring active surveillance (Table 3). These effects were larger among females, with the largest effect again observed in the preference for prostatectomy (Table 4). Compared with 17.5% (41/234) of those assigned to low information, 7.1% (17/240) of those assigned to high information preferred prostatectomy (risk difference = ‒10.4%, 95% CI = ‒16.3% to ‒4.6%).

The same pattern for combined label plus benefit-harm information effects was observed as for the primary outcome, with larger effects for *neoplasm* than for *lesion*. Among males, the largest effect was observed for preference for PSA monitoring (Table 5). Compared with 46.4% (104/224) of those assigned to *cancer* plus low information, 56.9% (132/232) of those assigned to *neoplasm* plus high information preferred PSA monitoring (risk difference = 10.5%, 95% CI = 1.3% to 19.6%). Among females, there were large effects on preference for prostatectomy and on preference for PSA monitoring (Table 6). Compared with 29.9% (70/234) of those assigned to *cancer* plus low information, 38.7% (89/230) of those assigned to *neoplasm* plus high information preferred PSA monitoring (risk difference = 8.8%, 95% CI = 0.2% to 17.4%).

#### Other secondary outcomes

Among males, there was no evidence of an effect of diagnostic label or benefit-harm information on other secondary outcomes: diagnosis anxiety, management choice anxiety, or choice to continue with conservative management after 5 years of stable results (among those initially choosing conservative management) (Tables 1, 3, and 5; Table S3). Among females, there was evidence that the combined effects of *neoplasm* plus high information and *lesion* plus high information decreased diagnostic anxiety (Table 6), with no evidence of other effects (Tables 2, 4, and 6).

## Discussion

This randomized hypothetical vignette experiment presents relevant empirical evidence to inform the ongoing discussion about changes in diagnostic labels within the pathology and clinical communities.^27^ Our findings support (1) potential patient preference for PSA monitoring as a “less is more” alternative to active surveillance; (2) development, evaluation, and implementation of patient information sheets or decision support tools for patients diagnosed with low-risk prostate disease; and (3) consideration of *neoplasm* as a new diagnostic label. Our study provides evidence to support the idea that relabeling low-risk prostate pathology findings is likely to reduce overtreatment.

We found that most Australian males receiving a hypothetical diagnosis of low-risk prostate disease would prefer conservative management, especially PSA monitoring, if offered. Although less frequent than in males, most female partners also preferred conservative management, especially active surveillance. Unlike previous studies, we included PSA monitoring as a “less is more” alternative to active surveillance and discovered that this option was very popular, especially among males. Prostate-specific antigen–based monitoring was the strategy evaluated in both the PIVOT^15-17^ and ProtecT^18-20^ trials, not the more intensive strategies used for active surveillance in current clinical practice, which use magnetic resonance imaging, biomarkers, and scheduled biopsies. This approach could be offered as an alternative to active surveillance to reduce treatment and monitoring harms, costs, and complexity.

Providing participants with detailed information about the absolute benefits and harms of the management options, including graphical display in the form of an icon array, increased preference for conservative management in both groups. This finding supports the development, evaluation, and implementation of patient information sheets or decision support tools for patients diagnosed with low-risk prostate disease, their partners, and their doctors. A recent RCT evaluated a decision aid tailored to the Australian health-care system (“Navigate”) that included males with a low-risk prostate cancer and their partners (allocated to the same randomly assigned arm together as dyads). The trial found that the decision aid resulted in higher uptake of active surveillance.^27^ A possible improvement to this and other tools, supported by our study findings, is to present multiple conservative management options, including PSA monitoring alone, as well as active surveillance.

Finally, we found that use of *neoplasm* instead of *cancer* in the diagnostic label also increased preference for conservative management, with the combination of both *neoplasm* and high information the most impactful intervention among those options tested (Figure 2). In contrast, use of the term *lesion* had minimal effect among males and inconsistent effects in female partners. Our study evaluated alternative diagnostic labels chosen by practicing clinician co-investigators (those treating patients with prostate cancer and those making the diagnosis) and patient and public co-investigators (with and without lived experience of prostate cancer).^13^ The intervention co-design aimed for labels acceptable to all end users that conveyed the low risk of disease progression.^28^ Our labels used less “medicalized” terms, which have been associated with a preference for less invasive management,^29^ effects that we observed with the use of *neoplasm* as a diagnostic label. The unexpected findings of a lack of reliable effects for the label *lesion* suggest that there may be limits to how “lay” the label should be to be perceived as legitimate. It is possible that *lesion* was perceived by some as a euphemism for cancer or that the term was simply not understood at all. This term did not seem to be a good alternative to the word *cancer* as the diagnostic label. A content analysis of data collected in free-text responses may provide further insights into how the 3 diagnostic labels were understood (manuscript in preparation).

Our findings are based on hypothetical scenarios, which limits applicability to decisions made in actual clinical practice. They may be broadly applicable, however, to Australians and people in other countries where conservative management for low-risk prostate cancer is common. Our study population included diversity in education (approximately 30% had a high school education or less) but was not ethnically or culturally diverse, and most participants lived in major cities. Of note, the management benefit-harm estimates provided in the high information groups were based on 1 RCT comparing observation vs prostatectomy conducted in the early PSA testing era (PIVOT^15-17^) and another RCT of males diagnosed based on a single PSA test and randomly assigned to PSA-based monitoring, prostatectomy, or RT (ProtecT^18-20^). There are no trial data evaluating active surveillance (as currently practiced), nor trials in males with prostate cancer detected with magnetic resonance imaging or by serial PSA screening, nor trials conducted in the era of effective pharmacological therapies for advanced disease. Thus, the benefit-harm estimates we used likely overestimate harms from disease spread and prostate cancer–specific mortality risks.

Two previous hypothetical randomized online experiments found beneficial effects for alternative diagnostic labels for low-risk prostate cancer. Hudnall et al.^30^ reported that *Prostate Cancer Gleason Group 1* decreased males’ preference for immediate definitive treatment and had lower ratings of anxiety compared with *Gleason Score 6* or *Indolent Lesion of Epithelial Origin*. Berlin et al.^22^ found strong preferences for *tumor* over *cancer* and for *prostatic acinar neoplasm of uncertain malignant potential* over *adenocarcinoma* among males with and without a history of prostate cancer and female partners. Like us, they found differences between males and female partners, with the latter more likely to prefer curative-intent treatment. This finding highlights the importance of communicating the low-risk nature of the pathology and benefits vs harms of management options to both the patient and his female partner.

Our study has limitations, most notably its hypothetical nature, which makes effects in real-world settings uncertain. The actual uptake of management strategies may differ to stated intentions by our study participants, who did not (or their partners did not) have prostate cancer. Our sample size calculation assumed no interaction between the effects of labels and information factors, but we found evidence that these effects were likely present. We may not have had sufficient power to detect clinically meaningful differences across the randomized combinations of label and information. Finally, our study design meant that we recruited only female partners; future research is needed to assess effects on male partner preferences.

## Conclusions

In this hypothetical vignette randomized experiment, the majority of Australian males and female partners preferred conservative management for low-risk prostate pathology findings, including less intensive monitoring than is commonly offered in practice. Clear communication of management benefits and harms and using *neoplasm* to describe low-risk pathology is likely to support conservative management choices and mitigate harms from overtreatment.

## Supplementary Material

djag248_Supplementary_Data

## Data Availability

The data underlying this article are available in the Open Science Framework osf.io/ruvs4.

## Supplementary material

Supplementary  is available at *Journal of the National Cancer Institute* online.
