# Supplementary material for: Impact of diagnostic labels and management benefit to harms information for low-risk prostate pathology: Two online factorial, randomized, hypothetical scenario experiments in Australian males and female partners
Source: J Natl Cancer Inst. 2026 Jul 24;118(11):2135–44. doi: 10.1093/jnci/djag248 (PMC13748740; doi:10.1093/jnci/djag248)
Supplement: Supplementary file 1 [file djag248_supplementary_data.docx]

**Supplementary Materials**

***Supplementary Boxes, Figure, Tables***

Box S1: Dignostic label interventions presented in the hypothetical scenarios

Box S2: Benefits-harms information interventions presented in the hypothetical scenarios

Box S3: Primary and secondary outcomes and covariates

Box S4: Estimands of interest

Figure S1: Additional information on absolute benefits and harms of management options provided in high information condition.

Table S1: Males - baseline characteristics of randomised groups: number (%)

Table S2: Female Partners - baseline characteristics of randomised groups: number (%)

Table S3: Diagnostic label effects in males across high information groups (Estimand 2: label effects with idealised practice comparator)

Table S4: Diagnostic label effects in female partners across high information groups (Estimand 2: label effects with idealised practice comparator)

***Supplementary Methods***

Survey

**Box S1: Dignostic label interventions presented in the hypothetical scenarios**

| The doctor has the biopsy test results and says: “we found a small focus of..” | |
| --- | --- |
| Control | **“low-risk prostate cancer, grade group 1”** |
| Alternative label 1 | **“low-risk prostate neoplasm”** |
| Alternative label 2 | **“low-risk prostate lesion”** |

**Box S2: Benefits-harms information interventions presented in the hypothetical scenarios**

| After giving you/your partner these test results, the doctor explains that there are four options. These are: | |
| --- | --- |
| Low information control | **Option 1. PSA monitoring:** where you/your partner keeps visiting the doctor to get check-ups and tests. Your partner will be monitored at regular points in time with PSA blood tests. This is to monitor the way the [diagnostic label] behaves. If it shows signs of growing, then treatment can be started.  **Option 2. Active surveillance:** where you/your partner keeps visiting the doctor toget check-ups and tests. Your partner will be monitored at regular points in time with PSA blood tests, MRI scans, and prostate biopsies. This is to monitor theway the [diagnostic label] behaves. If it shows signs of growing, then treatment can be started.  **Option 3. Radical prostatectomy:** where you/your partner has a surgical procedure to remove the prostate. This includes the [diagnostic label].  **Option 4. Radiotherapy:** where you/your partner has a non-surgical procedure on the prostate. The prostate is treated with radiation to destroy the [diagnostic label].  Studies have reported: For every 100 men found to have a [diagnostic label], 2 will die from this over the next 15 years, regardless of the treatment.  Different treatments have different advantages and disadvantages. |
| High information | **Text as above + iconarrays presented in Figure S1** |
|  | |

**Box S3: Primary and Secondary outcomes and covariates**

| **Primary Outcomes** |  |
| --- | --- |
| Choice of management approach: no immediate treatment (PSA monitoring or active surveillance) versus immediate treatment (prostatectomy, radiotherapy). | Direct choice between two management approaches (patient choices between the four management options grouped into two) |
| **Secondary Outcomes** |  |
| Management choice between PSA monitoring, active surveillance, prostatectomy, or radiotherapy. | Direct choice between specified options, one choice possible from four options. |
| Diagnosis anxiety. | Single-question Visual Analogue Scale (0-10)^1^ |
| Management choice anxiety. | Single-question Visual Analogue Scale (0-10)^1^ |
| Open-text explanation of management choice. | Free text (optional) |
| Participant management choice 5 years later (for those initially choosing PSA monitoring or active surveillance) | Direct choice between two management approaches: continue monitoring versus immediate treatment |
| **Covariates (potential confounders)** |  |
| Sociodemographic factors | Age, Gender, Partner status, Partner age, State or Territory location, Remoteness of residence (postcode), Health Insurance status, Education, Employment status, Household income, Indigenous status, Country of birth, Language spoken at home |
| Medical history | Personal history of cancer (non-prostate cancer), family history of prostate cancer, PSA testing history |
| General mood and wellbeing. | WHO (Five) Well-Being Questionnaire.^2^ |
| Medical minimiser/maximiser. | Single-Item Maximiser/Minimiser Elicitation Question (MM1).^3^ |
| Health literacy. | Single Item Literacy Screener (SILS).^4^ |
| Cancer worry. | Direct choice between specified options, one choice possible. |
| Self-efficacy. | Generalized Self-Efficacy Scale (GSE).^5^ |
| Notes  1. Davey HM, Barratt AL, Butow PN, Deeks JJ. A one-item question with a Likert or Visual Analog Scale adequately measured current anxiety. Journal of Clinical Epidemiology. 2007;60(4):356-360.  2. World Health Organization. Wellbeing measures in primary health care/the DepCare Project: report on a WHO meeting: Stockholm, Sweden, 12–13 February 1998. World Health Organization. Regional Office for Europe;1998.  3. Scherer LD, Zikmund-Fisher BJ. Eliciting medical maximizing-minimizing preferences with a single question: development and validation of the MM1. Medical Decision Making. 2020;40(4):545-550.  4. Morris NS, MacLean CD, Chew LD, Littenberg B. The Single Item Literacy Screener: evaluation of a brief instrument to identify limited reading ability. BMC family practice. 2006;7:1-7.  5. Schwarzer R, Jerusalem M. Generalized self-efficacy scale. J Weinman, S Wright, & M Johnston, Measures in health psychology: A user’s portfolio Causal and control beliefs. 1995;35:37. | |

**Box S4: Estimands of Interest**

**Estimand 1**. Effect of each alternative label compared with control label in the setting of low information provision (representing usual care). Using the multiarm analysis, we compared the Alternative label + Low information group to the Control label + Low information group.

**Estimand 2**. Effect of each alternative label compared with the control label in the setting of high information provision (representing idealised best practice). Using the multiarm analysis, we compared the Alternative label + High information group to the Control label + High information group.

**Estimand 3.** Effect of high information compared with low information in the setting of control label (representing usual care). Using the multiarm analysis, we compared the Control label + High information group to the Control label + Low information group.

**Estimand 4.** Combined effects of alternative label and high information (representing possible dual label and information intervention). Using the multiarm analysis, we compared the Alternative label + High information group to the Control label and Low information group.

**Figure S1: Additional information on absolute benefits and harms of management options provided in high information condition.**

Risk estimates are based on long-term follow-up in the PIVOT^1-3^ and ProtecT randomised controlled trials^4-6^. PSA, prostate-specific antigen

References:

1.	Wilt TJ, Brawer MK, Jones KM, et al. Radical Prostatectomy versus Observation for Localized Prostate Cancer. *New England Journal of Medicine.* 2012;367(3):203-213.

2.	Wilt TJ, Jones KM, Barry MJ, et al. Follow-up of Prostatectomy versus Observation for Early Prostate Cancer. *New England Journal of Medicine.* 2017;377(2):132-142.

3.	Wilt TJ, Vo TN, Langsetmo L, et al. Radical Prostatectomy or Observation for Clinically Localized Prostate Cancer: Extended Follow-up of the Prostate Cancer Intervention Versus Observation Trial (PIVOT). *Eur Urol.* 2020;77(6):713-724.

4.	Donovan Jenny L, Hamdy Freddie C, Lane JA, et al. Patient-Reported Outcomes 12 Years after Localized Prostate Cancer Treatment. *NEJM Evidence.* 2023;2(4):EVIDoa2300018.

5.	Hamdy FC, Donovan JL, Lane JA, et al. 10-Year Outcomes after Monitoring, Surgery, or Radiotherapy for Localized Prostate Cancer. *New England Journal of Medicine.* 2016;375(15):1415-1424.

6.	Hamdy FC, Donovan JL, Lane JA, et al. Fifteen-Year Outcomes after Monitoring, Surgery, or Radiotherapy for Prostate Cancer. *New England Journal of Medicine.* 2023;388(17):1547-1558.

**Sources for risk estimates used in the iconarray**

|  | Urinary Incontinence – 1+ pads per day (at 2 years) | Impotence - Erections not firm enough for intercourse (at 2 years) | Fecal leakage once or more per week (at 2 years) | Metastasis (at 15 years) | Definitive treatment (at 15 years) |
| --- | --- | --- | --- | --- | --- |
| PSA monitoring | 4%^3^ | 44%^1^ | 5%^3^ | 6.2%^4^ | 20.4%^2^ |
| AS | 4%^3^ | 53%^3^ | 5%^3^ | 6.2%^4^ | 61.1%^4^ |
| Prostatectomy | 26%^3^ | 81%^3^ | 4%^3^ | 3.0%^4^ | N/A |
| Radiotherapy | 4%^3^ | 66%^3^ | 10%^3^ | 3.3%^4^ | N/A |

1. PIVOT 2012 N Engl J Med 2012;367:203-13. DOI: 10.1056/NEJMoa1113162
2. PIVOT 2017 N Engl J Med 2017;377:132-42. DOI: 10.1056/NEJMoa1615869
3. ProtecT 2023 NEJM Evidence NEJM Evid 2023; 2 (4) <https://doi.org/10.1056/EVIDoa2300018>
4. ProtecT 2023 N Engl J Med 2023;388:1547-58. DOI: 10.1056/NEJMoa2214122 (15 years)

**Table S1: Males^1^ - Baseline characteristics of randomised groups: number (%)**

| **Characteristic** | **“Cancer”**  **+ Low Info (N=224)** | **“Cancer”**  **+ High Info (N=232)** | **“Neoplasm”**  **+ Low Info (N=214)** | **“Neoplasm”**  **+ High Info (N=232)** | **“Lesion”**  **+ Low Info (N=223)** | **“Lesion”**  **+ High Info (N=215)** |
| --- | --- | --- | --- | --- | --- | --- |
| Gender |  |  |  |  |  |  |
| Men | 215 (96%) | 218 (94%) | 201 (94%) | 221 (95%) | 219 (98%) | 210 (98%) |
| Other | 9 (4.0%) | 14 (6.0%) | 13 (6.1%) | 11 (4.7%) | 4 (1.8%) | 5 (2.3%) |
| Age |  |  |  |  |  |  |
| 50-59 | 80 (36%) | 72 (31%) | 64 (30%) | 76 (33%) | 74 (33%) | 63 (29%) |
| 60-69 | 78 (35%) | 85 (37%) | 84 (39%) | 83 (36%) | 77 (35%) | 91 (42%) |
| 70-79 | 51 (23%) | 66 (28%) | 55 (26%) | 66 (28%) | 56 (25%) | 50 (23%) |
| 80 or over | 15 (6.7%) | 9 (3.9%) | 11 (5.1%) | 7 (3.0%) | 16 (7.2%) | 11 (5.1%) |
| Indigenous | 7 (3.1%) | 1 (0.4%) | 6 (2.8%) | 5 (2.2%) | 4 (1.8%) | 6 (2.8%) |
| Country of birth |  |  |  |  |  |  |
| Australia | 165 (74%) | 179 (77%) | 164 (77%) | 168 (72%) | 169 (76%) | 159 (74%) |
| English language spoken at home | 218 (97%) | 220 (95%) | 207 (97%) | 221 (95%) | 211 (95%) | 207 (96%) |
| State or territory of residence |  |  |  |  |  |  |
| Australian Capital Territory | 2 (0.9%) | 2 (0.9%) | 4 (1.9%) | 3 (1.3%) | 7 (3.1%) | 1 (0.5%) |
| New South Wales | 71 (32%) | 83 (36%) | 60 (28%) | 55 (24%) | 67 (30%) | 70 (33%) |
| Northern Territory | 0 (0%) | 1 (0.4%) | 0 (0%) | 3 (1.3%) | 0 (0%) | 1 (0.5%) |
| Queensland | 56 (25%) | 49 (21%) | 41 (19%) | 56 (24%) | 52 (23%) | 53 (25%) |
| South Australia | 15 (6.7%) | 15 (6.5%) | 12 (5.6%) | 18 (7.8%) | 25 (11%) | 21 (9.8%) |
| Tasmania | 5 (2.2%) | 14 (6.0%) | 11 (5.1%) | 6 (2.6%) | 9 (4.0%) | 4 (1.9%) |
| Victoria | 49 (22%) | 53 (23%) | 55 (26%) | 63 (27%) | 47 (21%) | 51 (24%) |
| Western Australia | 26 (12%) | 15 (6.5%) | 31 (14%) | 28 (12%) | 16 (7.2%) | 14 (6.5%) |
| Rurality |  |  |  |  |  |  |
| metropolitan | 163 (73%) | 154 (67%) | 150 (70%) | 171 (75%) | 168 (75%) | 162 (76%) |
| rural | 60 (27%) | 77 (33%) | 64 (30%) | 57 (25%) | 55 (25%) | 51 (24%) |
| Health insurance | 121 (54%) | 126 (55%) | 121 (57%) | 113 (49%) | 124 (56%) | 123 (57%) |
| Partner |  |  |  |  |  |  |
| No Partner | 31 (14%) | 35 (15%) | 34 (16%) | 44 (19%) | 32 (14%) | 36 (17%) |
| Partnered | 163 (73%) | 177 (76%) | 154 (72%) | 156 (67%) | 161 (72%) | 146 (68%) |
| Previously Partnered | 30 (13%) | 20 (8.6%) | 26 (12%) | 32 (14%) | 30 (13%) | 33 (15%) |
| Education |  |  |  |  |  |  |
| High school or less | 71 (32%) | 75 (32%) | 67 (31%) | 80 (34%) | 65 (29%) | 76 (36%) |
| Non-University degree qualification | 74 (33%) | 78 (34%) | 86 (40%) | 89 (38%) | 79 (35%) | 72 (34%) |
| University degree | 77 (35%) | 79 (34%) | 60 (28%) | 63 (27%) | 79 (35%) | 65 (31%) |
| Employment |  |  |  |  |  |  |
| Employed | 104 (46%) | 104 (45%) | 105 (49%) | 105 (45%) | 109 (49%) | 103 (48%) |
| Not in workforce | 104 (46%) | 112 (48%) | 101 (47%) | 115 (50%) | 103 (46%) | 98 (46%) |
| Unemployed | 16 (7.1%) | 16 (6.9%) | 8 (3.7%) | 12 (5.2%) | 11 (4.9%) | 14 (6.5%) |
| Income |  |  |  |  |  |  |
| less than $100,000 | 147 (69%) | 151 (69%) | 145 (73%) | 155 (70%) | 151 (70%) | 143 (70%) |
| $100-200,000 | 52 (24%) | 60 (28%) | 44 (22%) | 56 (25%) | 52 (24%) | 51 (25%) |
| >$200,000 | 14 (6.6%) | 7 (3.2%) | 11 (5.5%) | 10 (4.5%) | 13 (6.0%) | 9 (4.4%) |
| Health literacy |  |  |  |  |  |  |
| Adequate | 195 (87%) | 206 (89%) | 193 (90%) | 203 (88%) | 194 (87%) | 189 (88%) |
| Limited | 29 (13%) | 26 (11%) | 21 (9.8%) | 29 (13%) | 29 (13%) | 26 (12%) |
| Perceived Health status |  |  |  |  |  |  |
| Healthy | 156 (70%) | 150 (65%) | 156 (73%) | 143 (62%) | 145 (65%) | 146 (68%) |
| Personal cancer diagnosis  (not prostate cancer) | 39 (17%) | 29 (13%) | 23 (11%) | 32 (14%) | 32 (14%) | 35 (16%) |
| Family history of any cancer (non-skin cancer) | 90 (41%) | 115 (51%) | 95 (45%) | 104 (46%) | 98 (44%) | 87 (42%) |
| Family history of prostate cancer | 22 (9.8%) | 20 (8.6%) | 24 (11%) | 28 (12%) | 18 (8.1%) | 14 (6.5%) |
| Previously had a PSA test | 144 (64%) | 130 (56%) | 135 (63%) | 138 (59%) | 149 (67%) | 128 (60%) |
| Worry about prostate cancer |  |  |  |  |  |  |
| Not worried at all | 74 (33%) | 76 (33%) | 65 (30%) | 66 (28%) | 78 (35%) | 73 (34%) |
| A bit worried | 125 (56%) | 117 (50%) | 123 (57%) | 130 (56%) | 123 (55%) | 113 (53%) |
| Very/Quite worried | 25 (11%) | 39 (17%) | 26 (12%) | 36 (16%) | 22 (9.9%) | 29 (13%) |
| Maximiser (on minimiser-maximiser scale) | 128 (57%) | 123 (53%) | 130 (61%) | 127 (55%) | 129 (58%) | 123 (57%) |
| Wellbeing (rescaled to 0 to 100) (median, Q1, Q3)^2^ | 64 (40, 76) | 64 (44, 78) | 64 (44, 76) | 60 (38, 76) | 64 (40, 80) | 64 (40, 76) |
| Self-efficacy (rescaled to 0 to 100) (median, Q1, Q3)^3^ | 75 (73, 83) | 78 (70, 86) | 75 (73, 83) | 75 (70, 85) | 75 (73, 85) | (73, 88) |

Notes

1. Includes other people with a prostate not identifying as a man
2. Higher scores indicate more wellbeing

2. Higher scores indicate more self-efficacy

**Table S2: Female Partners^1^ - Baseline characteristics of randomised groups: number (%)**

| **Characteristic** | **“Cancer” + Low Info (N=234)** | **“Cancer” + High Info (N=240)** | **“Neoplasm” + Low Info (N=236)** | **“Neoplasm” + High Info (N=230)** | **“Lesion” + Low Info (N=247)** | **“Lesion” + High Info (N=248)** |
| --- | --- | --- | --- | --- | --- | --- |
| Gender |  |  |  |  |  |  |
| Women | 234 (100%) | 240 (100%) | 234 (99%) | 229 (100%) | 245 (99%) | 247 (100%) |
| Other | 0 (0%) | 0 (0%) | 2 (0.8%) | 1 (0.4%) | 2 (0.8%) | 1 (0.4%) |
| Age |  |  |  |  |  |  |
| 30-39 | 15 (6.4%) | 19 (7.9%) | 4 (1.7%) | 14 (6.1%) | 11 (4.5%) | 15 (6.0%) |
| 40-49 | 45 (19%) | 38 (16%) | 41 (17%) | 46 (20%) | 49 (20%) | 36 (15%) |
| 50-59 | 71 (30%) | 76 (32%) | 86 (36%) | 66 (29%) | 75 (30%) | 77 (31%) |
| 60-69 | 66 (28%) | 61 (25%) | 71 (30%) | 65 (28%) | 70 (28%) | 82 (33%) |
| 70-79 | 33 (14%) | 45 (19%) | 32 (14%) | 37 (16%) | 41 (17%) | 36 (15%) |
| 80 or over | 4 (1.7%) | 1 (0.4%) | 2 (0.8%) | 2 (0.9%) | 1 (0.4%) | 2 (0.8%) |
| Indigenous | 3 (1.3%) | 3 (1.3%) | 7 (3.0%) | 0 (0%) | 5 (2.0%) | 7 (2.8%) |
| Country of birth |  |  |  |  |  |  |
| Australia | 167 (71%) | 180 (75%) | 176 (75%) | 160 (70%) | 188 (76%) | 180 (73%) |
| English language spoken at home | 222 (95%) | 231 (96%) | 221 (94%) | 218 (95%) | 240 (97%) | 236 (95%) |
| State or territory of residence |  |  |  |  |  |  |
| Australian Capital Territory | 1 (0.4%) | 4 (1.7%) | 1 (0.4%) | 2 (0.9%) | 2 (0.8%) | 5 (2.0%) |
| New South Wales | 76 (32%) | 65 (27%) | 92 (39%) | 68 (30%) | 92 (37%) | 85 (34%) |
| Northern Territory | 1 (0.4%) | 2 (0.8%) | 1 (0.4%) | 2 (0.9%) | 0 (0%) | 0 (0%) |
| Queensland | 38 (16%) | 51 (21%) | 46 (19%) | 30 (13%) | 47 (19%) | 41 (17%) |
| South Australia | 20 (8.5%) | 12 (5.0%) | 16 (6.8%) | 16 (7.0%) | 16 (6.5%) | 11 (4.4%) |
| Tasmania | 8 (3.4%) | 7 (2.9%) | 3 (1.3%) | 11 (4.8%) | 4 (1.6%) | 9 (3.6%) |
| Victoria | 71 (30%) | 69 (29%) | 58 (25%) | 74 (32%) | 62 (25%) | 76 (31%) |
| Western Australia | 19 (8.1%) | 30 (13%) | 19 (8.1%) | 27 (12%) | 24 (9.7%) | 21 (8.5%) |
| Rurality |  |  |  |  |  |  |
| metropolitan | 172 (74%) | 182 (77%) | 186 (79%) | 165 (73%) | 182 (74%) | 179 (73%) |
| rural | 60 (26%) | 55 (23%) | 48 (21%) | 62 (27%) | 63 (26%) | 67 (27%) |
| Health insurance | 133 (57%) | 152 (64%) | 136 (58%) | 135 (59%) | 133 (54%) | 142 (57%) |
| Education |  |  |  |  |  |  |
| High school or less | 59 (25%) | 78 (33%) | 71 (30%) | 65 (28%) | 66 (27%) | 76 (31%) |
| Non-University degree qualification | 94 (40%) | 72 (30%) | 84 (36%) | 82 (36%) | 91 (37%) | 87 (35%) |
| University degree | 81 (35%) | 89 (37%) | 79 (34%) | 82 (36%) | 88 (36%) | 84 (34%) |
| Employment |  |  |  |  |  |  |
| Employed | 133 (57%) | 125 (52%) | 124 (53%) | 128 (56%) | 130 (53%) | 128 (52%) |
| Not in workforce | 91 (39%) | 96 (40%) | 100 (42%) | 84 (37%) | 105 (43%) | 107 (43%) |
| Unemployed | 10 (4.3%) | 18 (7.5%) | 12 (5.1%) | 17 (7.4%) | 12 (4.9%) | 13 (5.2%) |
| Income |  |  |  |  |  |  |
| less than $100,000 | 124 (58%) | 133 (60%) | 138 (64%) | 132 (63%) | 148 (64%) | 127 (56%) |
| $100-200,000 | 71 (33%) | 72 (33%) | 59 (27%) | 64 (30%) | 68 (29%) | 81 (36%) |
| >$200,000 | 18 (8.5%) | 16 (7.2%) | 20 (9.2%) | 14 (6.7%) | 17 (7.3%) | 18 (8.0%) |
| Health literacy |  |  |  |  |  |  |
| Adequate | 215 (92%) | 212 (88%) | 211 (89%) | 207 (90%) | 230 (93%) | 220 (89%) |
| Limited | 19 (8.1%) | 28 (12%) | 25 (11%) | 23 (10%) | 17 (6.9%) | 28 (11%) |
| Perceived Health status |  |  |  |  |  |  |
| Healthy | 183 (78%) | 187 (78%) | 182 (77%) | 195 (85%) | 211 (85%) | 189 (76%) |
| Personal cancer diagnosis | 36 (15%) | 21 (8.8%) | 34 (15%) | 24 (10%) | 36 (15%) | 27 (11%) |
| Family history of cancer (non-skin keratinocyte cancer) | 97 (42%) | 112 (47%) | 117 (51%) | 115 (50%) | 121 (50%) | 124 (51%) |
| Family history of prostate cancer | 17 (7.3%) | 23 (9.6%) | 24 (10%) | 21 (9.1%) | 21 (8.5%) | 31 (13%) |
| Partner has previously had a PSA test | 100 (43%) | 114 (48%) | 120 (51%) | 105 (46%) | 124 (50%) | 124 (50%) |
| Worry about prostate cancer |  |  |  |  |  |  |
| Not worried at all | 54 (23%) | 60 (25%) | 53 (22%) | 57 (25%) | 54 (22%) | 47 (19%) |
| A bit worried | 135 (58%) | 136 (57%) | 141 (60%) | 129 (56%) | 151 (61%) | 152 (61%) |
| Very/Quite worried | 45 (19%) | 44 (18%) | 42 (18%) | 44 (19%) | 42 (17%) | 49 (20%) |
| Maximiser (on minimiser-maximiser scale) | 153 (65%) | 147 (61%) | 156 (66%) | 145 (63%) | 167 (68%) | 157 (63%) |
| Wellbeing (rescaled to 0 to 100) (median, Q1, Q3)^2^ | 56 (36, 76) | 56 (32, 72) | 56 (32, 76) | 60 (40, 72) | 60 (36, 72) | 56 (36, 72) |
| Self-efficacy (rescaled to 0 to 100) (median, Q1, Q3)^3^ | 75 (70, 83) | 75 (68, 80) | 75 (70, 80) | 75 (70, 83) | 75 (70, 80) | 75 70, 83) |

Notes:

1. Includes other people without a prostate not identifying as a woman
2. Higher scores indicate more wellbeing
3. Higher scores indicate more self-efficacy

**Table S3: Diagnostic label effects in males across high information groups (Estimand 2: label effects with idealised practice comparator)**

| **Outcome** | **Prostate cancer**  **N=232** | **Prostate neoplasm**  **N=232** | **Absolute Difference (95% CI)^1^** | **Prostate lesion**  **N=215** | **Absolute Difference (95% CI)^1^** |
| --- | --- | --- | --- | --- | --- |
| Prefer curative-intent treatment | 29 (12.5%) | 17 (7.3%) | -5.2%  (-10.6%, 0.2%)^2^ | 33 (15.3%) | +2.8 %  (-3.6%, 9.2%)^3^ |
| Prefer prostatectomy | 12 (5.2%) | 10 (4.3%) | -0.9%  (-4.7%, 3.0%) | 18 (8.4%) | 3.2%  (-1.5%, 7.9%) |
| Prefer radiotherapy | 17 (7.3%) | 7 (3%) | -4.3%  (-8.3%,  -0.3%) | 15 (7%) | -0.4%  (-5.1%, 4.4%) |
| Prefer active surveillance | 79 (34.1%) | 83 (35.8%) | +1.7%  (-7.0%, 10.4%) | 81 (37.7%) | +3.6%  (-5.3%, 12.5%) |
| Prefer PSA monitoring | 124 (53.4%) | 132 (56.9%) | +3.4%  (-5.6%, 12.5%) | 101 (47%) | -6.5%  (-15.7%, 2.8%) |
| Diagnosis anxiety (visual analogue scale 1 to 10) (mean, SD) | 4.59 (2.65) | 4.78 (2.68) | MD: 0.19  (-0.30, 0.68) | 4.67 (2.74) | MD: 0.20  (-0.22, 0.63) |
| Management anxiety (visual analogue scale 1 to 10) (mean, SD) | 4.16 (2.60) | 4.22 (2.55) | MD: 0.06  (-0.41, 0.53) | 4.11 (2.73) | MD: -0.048  (-0.54, 0.45) |
| After 5 years of surveillance/monitoring choose curative surgery without medical indication^4^ | 24 (11.8%) | 25 (11.6% | -0.2% (-6.4-6%) | 16 (8.8%) | -3% (-9.1-3.1%) |

Notes:

1. RD = risk difference; MD = mean difference

2. P value =0.05 for ‘prostate neoplasm’ label effect on preference for curative-intent treatment in multivariable regression model

3. P value -0.28 for ‘prostate lesion’ label effect on preference for curative-intent treatment in multivariable regression model

4. Percentages are for those who did not choose initial curative treatment (i.e. denominators are those who chose active surveillance or PSA monitoring)

**Table S4: Diagnostic label effects in females across high information groups (Estimand 2: label effects with idealised practice comparator)**

| **Outcome** | **Prostate cancer**  **N=240** | **Prostate neoplasm**  **N=230** | **Absolute Difference (95% CI)** | **Prostate lesion**  **N=248** | **Absolute Difference (95% CI)** |
| --- | --- | --- | --- | --- | --- |
| Prefer curative-intent treatment | 41 (17.1%) | 34 (14.8%) | -2.3%  (-8.9, 4.3%) | 46 (18.5%) | 1.4%  (-5.4. 8.2%) |
| Prefer prostatectomy | 17 (7.1%) | 17  (7.4%) | 0.3%  (-4.4%, 5.0%) | 26 (10.5%) | 3.4%  (-1.6%, 8.4%) |
| Prefer radiotherapy | 24 (10%) | 17  (7.4%) | -2.6%  (-7.7%, 2.5%) | 20 (8.1%) | -1.9%  (-7.0%. 3.1%) |
| Prefer active surveillance | 118 (49.2%) | 107 (46.5%) | -2.6%  (-11.7%, 6.4%) | 108 (43.5%) | -5.6%  (-14.4%, 3.2%) |
| Prefer PSA monitoring | 81 (33.8%) | 89 (38.7%) | 5.0%  (-3.7%, 13.6%) | 94 (37.9%) | 4.2%  (-4.3%, 12.6%) |
| Diagnosis anxiety (visual analogue scale 1 to 10) (mean, SD)^4^ | 5.84 (2.43) | 5.47  (2.48) | -0.37  (-0.82, 0.073) | 5.84 (2.22) | -0.003  (-0.42, 0.41) |
| Management anxiety (visual analogue scale 1 to 10) (mean, SD)^5^ | 5.40  (2.49) | 4.94  (2.53) | -0.46  (-0.92, -0.007) | 5.24 (2.29) | -0.16  (-0.59, 0.26) |
| After 5 years of surveillance/monitoring choose curative surgery without medical indication^6^ | 26 (13.1%) | 20 (10.2%) | -2.9%  (-9.2%, 3.4%) | 32 (15.8%) | 2.7%  (-4.2%, 9.6%) |

Notes:

1. RD = risk difference; MD = mean difference

2. P value =0.60 for ‘prostate neoplasm’ label effect on preference for curative-intent treatment in multivariable regression model

3. P value =0.92 for ‘prostate lesion’ label effect on preference for curative-intent treatment in multivariable regression model

4. Missing outcome for one person in prostate cancer group and one person in prostate lesion group

5. Missing outcome for one person in the prostate lesion group

6. Percentages are for those who did not choose initial curative treatment (i.e. denominators are those who chose active surveillance or PSA monitoring)
